# Abolish the Use of Liquors

**Published:** 1881-07

**Authors:** 


### ABOLISH THE USE OF LIQUORS.

### GOOD REASONS FOR DOING AWAY WITH ALCOHOLIC DRINKS.

[From an Exchange.]

They deprive men of their reason for the time being.

They destroy men of the greatest intellectual strength.

They foster and encourage every species of immorality.

They bar the progress of civilization and religion.

They destroy the peace and happiness of tens of thousands of families.

They reduce many virtuous wives and children to poverty.

They cause many thousands of murders.

They prevent all reformation of character.

They render abortive the strongest resolutions.

The millions of property expended are lost.

They cause the majority of the cases of insanity.

They destroy both the body and the soul.

They burden sober people with millions for the support of paupers.

They cause immense expenditure to prevent crime.

They cost sober people immense sums in charity.

They burden the country with immense taxes.

Because moderate drinkers want the temptation removed.

Drunkards want the opportunity removed.

Sober people want the nuisance removed.

Taxpayers want the burden removed.

The prohibition would save thousands now falling.

The sale exposes our families to destruction.

The sale exposes our persons to insult.

The sale upholds the vicious and idle at the expense of the industrious and virtuous.

The sale subjects the sober to great oppression.

It takes the sober man's earnings to support the drunkard.

It subjects numberless wives to untold sufferings.

It is contrary to the Bible.

It is contrary to common sense.

We have a right to rid ourselves of the burden.

---

Toddlekins is a very small man, indeed, but he said he never minded it at all until his three boys began to grow tall, strapping young fellows and his wife began to cut down their old clothes to fit him. And then he said he did get mad.

